# Optical Detection of Dangerous Road Conditions

**DOI:** 10.3390/s19061360

**Published:** 2019-03-19

**Authors:** Armando Piccardi, Lorenzo Colace

**Affiliations:** NooEL—Nonlinear Optics and OptoElectronics Laboratory and CNIT—Photonic Networks and Technologies National Lab, Department of Engineering, University ROMA TRE, Via Vito Volterra 62, 00146 Rome, Italy; lorenzo.colace@uniroma3.it

**Keywords:** optical sensor, scattering from rough surfaces, polarized scattering, polarization contrast ratio

## Abstract

We demonstrated an optical method to evaluate the state of asphalt due to the presence of atmospheric agents using the measurement of the polarization/depolarization state of near infrared radiation. Different sensing geometries were studied to determine the most efficient ones in terms of performance, reliability and compactness. Our results showed that we could distinguish between a safe surface and three different dangerous surfaces, demonstrating the reliability and selectivity of the proposed approach and its suitability for implementing a sensor.

## 1. Introduction

The assessment of surface conditions that are exposed to the effects of atmospheric agents is crucial when dealing with safety, requiring the development of technologies to exploit sensing elements to assess and reduce the associated risks.

For instance, a number of methods have been developed for ice detection, each focused on a specific application from aircraft safety to cooling systems or environmental monitoring: these exploit combined measurements of temperature and humidity [1,2,3], capacitive [4,5,6] or piezoelectric [7] sensors and fiber optic devices [8,9], and some of them are available as commercial systems [10]. Contact sensors for ice detection on roads or other surfaces are moderately reliable, but they have problems in terms of installation and maintenance, since they need to be mounted directly on the iced surface. For these reasons, contactless systems based on electrical resistance measurements [11], reflected radiation at microwaves [12] or optical frequencies [13], and infrared imaging [14,15] have been proposed.

With reference to road safety, smart systems equipped on both vehicles and the roadside are desirable when there is the need for a reliable way to distinguish between safe and potentially dangerous driving situations [16,17].

A number of methods have been developed based on the refractive index difference of asphalt, water and ice, by measuring light reflection at different wavelengths. Unfortunately, they show limited reliability due to their low sensitivity, thus preventing selective measurement [18,19,20]. More recently it has been demonstrated that more information can be derived from the analysis of the reflected polarization, which is very sensitive to different environmental conditions [21,22,23,24].

Here we propose an optical method to detect different surface conditions based on the measurement of two orthogonally polarized components of a light beam scattered by the surface. Since the polarization of light is sensitive to the surface under investigation, its analysis gives information on the surface itself and it allows to distinguish different conditions.

This method was proposed in recent paper where the working principle and some preliminary results were reported [25]. It was based on the combined measurement of the polarization contrast ratio and the amplitude of the scattered light, and it focused on the identification of ice on an asphalt surface.

In this work, we started with the original idea and its preliminary results and developed an optical method to distinguish different surface conditions. Even though, in principle, this method is not dependent on the type of surface, our work focused on detecting the state of an asphalt surface and assessing potentially dangerous road conditions.

Using polarimetry measurement, we investigated different sensing configurations in order to optimize both reliability and selectivity in the detection of asphalt conditions. Furthermore, we implemented a sensing device and developed an electronic read-out suitable to handle the optical signals.

## 2. Materials and Methods

To investigate the different surfaces we employed a diode that emitted at λ = 980 nm. A near infrared source has several advantages: first of all, it is invisible to human eye and therefore it avoids the risk of annoying light scattering; it is in a wavelength range where the most common atmospheric agents (e.g., water and ice) feature a high degree of selectivity [26]; and, additionally, low cost near infrared optoelectronic devices are widely available. The radiation is modulated with a square wave at a frequency of about 1 kHz, which optimizes the detection sensitivity and immunity to environmental (i.e., continuous) light when a narrowband receiver is used.

**Figure 1:**
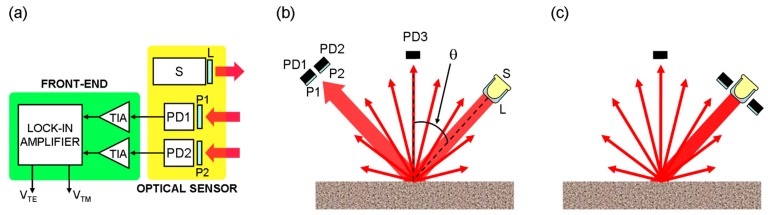
Block diagram and set-up. (**a**) Block diagram of the sensor. The radiation from the LED (S) was collimated by a lens (L) and impinged on the surface under investigation. After being reflected/scattered, it was detected by two photodiodes (PD1 and PD2). Two polarizers (P1 and P2) selected the horizontal and vertical polarization components. Two trans-impedance amplifiers (TIA) and the lock-in amplifier converted the optical signals into two voltage levels ready for the signal processing. (**b**) θ–2θ configuration. The incidence angle θ was referred to the surface normal and a third photodiode (PD3) was used to measure the unpolarized scattering. (**c**) Backscattering configuration. The set-up was analogous to (**b**), but both photodetectors collecting the polarized scattering were mounted on the same side of the source.

A lens was used to collimate the radiation and obtain a spot size of about 5 mm on the surface under investigation. This solution compensates for beam diffraction, allowing maximization of the light impinging on the detectors. A polarizer can be added to the source to study the sensor response as a function of incident light polarization. Two further polarizers were used to select the polarization components of the scattered light parallel and perpendicular to the incident plane and a couple of photodiodes detected polarized radiation and converted the optical signals into current signals. The front-end electronics performed a current to voltage conversion by means of trans-impedance amplifiers (TIA). The signals corresponding to the orthogonal polarizations were detected by a heterodyne receiver (i.e., a lock-in amplifier) in order to extract and process the information about the surface condition even in the presence of large noise. The block diagram of the sensor is sketched in Figure 1a.

The figure of merit that was exploited to distinguish between different surfaces was the polarization contrast ratio (PC). This parameter is a normalized quantity sensitive to the differences between the polarization components, which was defined as PC = (I_TE_ − I_TM_)/(I_TE_ + I_TM_), where I_TE/TM_ is the light intensity corresponding to the two polarization components. In addition, the setup was equipped with a third photodetector in order to measure the total unpolarized light scattered from the surface, thus providing a second useful parameter.

Since Fresnel’s coefficients depend on the incidence angle [27], preliminary measurements were performed to identify the optimal geometric configuration. Thus, we first found the angle for which the distance among the polarization contrast values corresponding to different surface states was maximized. Once this angle was found, the polarization contrast was calculated from the converted voltage values (representing the light intensities) and it was plotted against the total (unpolarized) scattered light. In this way, the position of the single point on the parameter space (PC vs unpolarized scattering) is a function of the surface condition [25].

**Figure 2:**
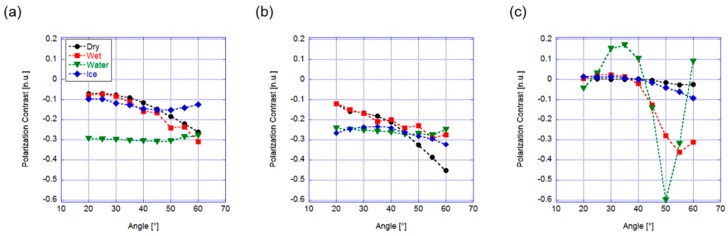
θ–2θ configuration. Polarization contrast versus incidence angle for (**a**) unpolarized, (**b**) horizontally and (**c**) vertically polarized incident light.

**Figure 3:**
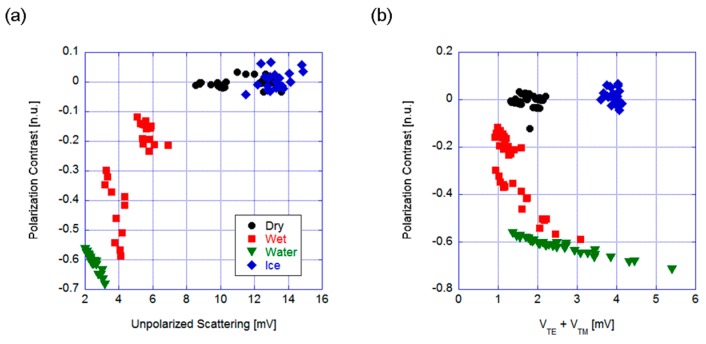
Mirror-like configuration. Polarization contrast versus (**a**) the total scattering and (**b**) the sum of the two orthogonal polarized components (V_TE_ and V_TM_, the voltage corresponding to horizontal and vertical polarization, respectively).

**Figure 4:**
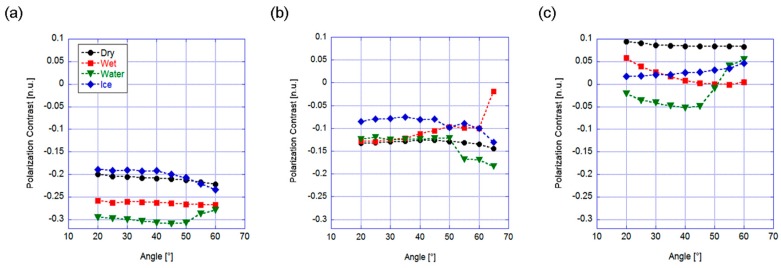
Backscattering configuration. Polarization contrast versus incidence angle for (**a**) unpolarized, (**b**) horizontally and (**c**) vertically polarized incident light.

**Figure 5:**
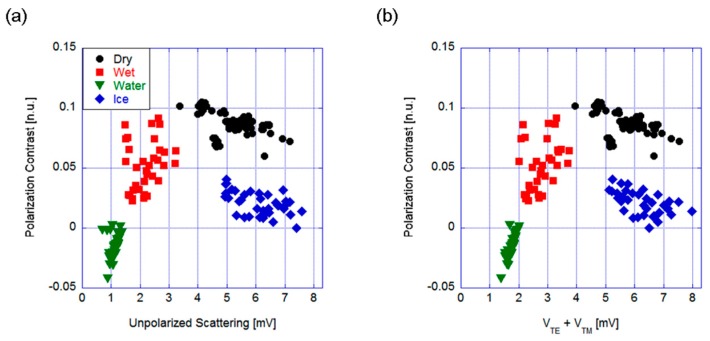
Backscattering configuration. Polarization contrast versus (**a**) the total scattering and (**b**) the sum of the two orthogonal polarized components.

We focused our experiments on asphalt surfaces with the aim of detecting different atmospheric agents affecting road safety. The investigated samples refer to different asphalt conditions, as indicated in Figure 2, Figure 3, Figure 4 and Figure 5 (later in the text). Four kinds of surfaces were tested. The reference condition corresponds to dry asphalt, which can be identified as a safe condition. Several kinds of asphalts were tested, differing in both roughness and composition. Though no formal classification of the kinds of asphalt was performed, we verified (using data in Figure 3 and Figure 5, see Section 3) that all kinds of asphalt shared similar optical properties with respect to the scattered polarized radiation.

A first potentially dangerous condition is the presence of water on the asphalt surface. This has been studied in two different states: wet asphalt and asphalt where the surface was completely covered by a thick layer (i.e., a few millimetres) of water. A second hazardous condition was determined by the presence of ice: we used an asphalt surface that was covered by a thin ice layer (1 mm).

Thus, we investigated the scattered radiation from four different states, identified as dry, wet, water and ice in the following. We would like to emphasize that it is not within the scope of this paper to investigate the optical properties of these kinds of surfaces, but only to validate the approach to efficiently detect the surface condtions.

Two major geometries were investigated, as shown in Figure 1. The first was based on Fresnel reflection with the source and detectors mounted in a θ–2θ configuration (Figure 1b). Using this geometry, the horizontal (vertical) polarized reflected radiation was maximized (minimized) at the Brewster angle (like for a smooth surface) and thus featured a wide range of polarization contrast ratio with benefits to the system sensitivity. Nevertheless, the system required the source (S) and the detector (D) to be placed in different positions, increasing the space needed for the sensor.

The alternative geometry measured the polarization components of the backscattered light that was always present during scattering from a rough surface (Figure 1c). In this case, higher sensitivity was needed from the receiver, but the resulting sensor was much more compact. In both configurations, the presence of an additional detector, used to sense the unpolarized scattered light from the direction of the surface normal, was considered as well.

The experimental set-up was realized with commercial components: the source was a LED940E (Thorlabs) with a maximum drive current of 100 mA for a power of 18 mW. The polarizers were LPV050 (Thorlabs) with an extinction ratio higher than 100000:1 at the employed wavelength and the detectors were BPW34F (OSRAM) that worked in the 780–1100 nm spectral range and exhibited a maximum responsivity of 0.7 A/W at λ = 950 nm and a sensitive area of 2.65 × 2.65 mm^2^.

## 3. Results

In the first geometry, Figure 1b, the source and the detectors were mounted on two arms in a goniometric system to preserve the symmetry of the incident and reflection angles, with the source mounted approximately 30 cm from the surface under investigation. A preliminary measurement involved the evaluation of the polarization contrast as a function of the incident angle [28]. The results in the case of unpolarized light from the source are shown in Figure 2a, after being averaged over several acquisitions. Most of the curves corresponding to the different kind of surfaces were superposed, preventing the identification of the asphalt conditions. Only for θ = 50° were the curves separated, but the polarization contrast range was small. We repeated the same measurement using polarized light, exploiting the polarization dependence of the Fresnel coefficients. Figure 2b,c reports on the angular dependence of the scattered light from a source with horizontal and vertical polarization, respectively. When using horizontal polarization from the source (i.e., perpendicular to the plane of incidence) the curves exhibited some angles (θ > 50°) where the safe condition was separated from all the others, but none of the “dangerous” states were clearly distinguishable. On the other hand, the vertical polarized light (i.e., polarization parallel to the plane of incidence) guaranteed a larger separation between the different conditions, at least around 50°, where the polarization contrast value associated with water (for both the water layer and wet cases) exhibited a minimum. This was due to the Brewster angle associated to the water surface (approximately 53°). The transmitted radiation was minimized, increasing the intensity associated with the reflected radiation, and thus increasing the absolute value of the polarization contrast ratio. Thus, we concluded that vertical polarized radiation offered the best performances for the realization of a sensor in this geometry.

After setting the vertically polarized source to 50° with respect to the surface normal, we measured the intensities associated with the two polarization components and calculated the polarization contrast for all the surfaces under investigation. A large number of acquisitions were taken in order to evaluate statistical errors and to simulate the “on field” conditions, including motion and different kinds of asphalt surfaces.

To evaluate the surface state, we built two parameter planes. The first included the polarization contrast values versus the total unpolarized scattering, evaluated from the measurement of the light scattered along the surface normal and collected by the third photodetector. The second was composed of the polarization contrast versus the sum of the two polarization components. In this way, each surface condition could be defined by a sector of the parameter plane, as the values of the experimental measurements corresponding to the different surfaces generated a group of points (with the point dispersion being due to measurement uncertainty, the different kinds of asphalt, or slightly different surface conditions, like water or ice layer thickness), which should be included in the corresponding part of the plane.

Figure 3 shows the graphs obtained for a vertical polarized source and incidence angle of 50°. The former case, PC versus unpolarized scattering (Figure 3a), exhibited a problem with the dry (i.e., safe) and iced (i.e., dangerous) conditions, where the two groups of points were partially overlapping. Thus, the choice of geometry and measured parameters did not allow selective measurement for the considered surface states. Conversely, using the sum of the orthogonal polarizations allowed us to discriminate among all four different states even if the voltage signal levels were slightly lower (thus, the required sensitivity was higher), as shown in Figure 3b. Even if the water and wet conditions were very close, they almost correspond to the same kind of dangerous conditions, thus the risk of confusing the two surfaces is not critical and it would not affect the performance of the sensor in an application-based investigation.

The polarization contrast was close to zero for both the dry asphalt and the ice cases, which indicates that the scattered light was almost unpolarized, probably due to the depolarization effects from the high surface roughness in both cases. The presence of water enhanced light polarization, even if the groups of points corresponding to the wet and water layer were quite dispersed. Nevertheless, this geometry allows us to discriminate among some potentially dangerous conditions. Furthermore, the third photodiode is not necessary.

We performed the same kind of measurement procedure in the backscattering configuration. As for the former geometry, we first verified which angle maximized the separation between different conditions, as sketched in Figure 4, for the unpolarized (Figure 4a) and polarized (horizontally and vertically, Figure 4b,c, respectively) incident radiation.

It can be noted that the polarization contrast was lower with respect to the previous case. This was expected considering the lower signal level due to different geometry. Nevertheless, here the four road conditions often exhibited quite different values. In the unpolarized case the whole angle range below θ = 40° could be used, even if the dry condition was always close to the ice curve. When examining the horizontal polarized case, we can easily see that the curves were superposed for almost all the angles (except for θ = 65°, where dry and ice conditions were still too close to ensure reliable detection), thus this case was also not taken into consideration. The vertical polarized light case provided much better results, exhibiting several angles where all the curves were well separated. In particular, at θ = 20° the graph shows almost equal distances among the curves in a quite large interval of about 0.12 for polarization contrast. As a further advantage with respect to the unpolarized case, the dry condition had the highest contrast value, and it was well separated from all the other potentially dangerous situations. In addition, in this case, the vertical polarized radiation was considered to be the best choice to discriminate among the different asphalt conditions.

Thus, once the incident angle was set to about 20°, we measured the two polarized scattering components. The results are shown in Figure 5, with the polarization contrast as a function of both the total scattering (Figure 5a) and the sum of the signals corresponding to the two orthogonal polarizations (Figure 5b).

Remarkably, the two graphs were very similar, which made the third photodiode unnecessary. In other words, the backscattered light contained all the information needed, allowing us to reduce the sensor complexity.

The dry asphalt had the highest value of both contrast and total scattering, thus defining a region of safe condition in the parameter space, while the polarization contrast easily allowed the detection of the presence of ice. The water layer showed the lower value of (back)scattering due to the smooth surface, while the group of points corresponding to the wet condition was in the middle between water and dry, as expected. The results for each group of points were well separated from the others with reasonable dispersion occurring, which allowed us to divide the parameter space into four parts that were associated to the different asphalt conditions. For example, PC = 0.05 would divide the dry asphalt and the ice states.

Hence, the geometry that employed the backscattered radiation guaranteed the best performance. Moreover, employing only two photodiodes mounted on the same side of the source allowed for the most compact configuration for the practical realization of sensors based on this method.

## 4. Discussion

The presented results demonstrate the possibility of implementing an optical sensor to distinguish several asphalt conditions. The information on the state is given by the position of the measured values on the parameter plane: for instance, Figure 4 suggests that the plane can be divided into four parts corresponding to the four different states. The edges of the four parts define threshold values of the polarization contrast and the sum of the components, which could be represented by straight lines. In other words, our results can be used as a calibration of the system. A sensor employing this method will measure the suitable quantities by placing the corresponding point on the parameter plane. The surface state will be identified by comparing the position of the measured point with the calculated thresholds.

Future developments of our work will encompass the increased number of detected conditions, including a study of the perturbations coming from disturbances such as the presence of dust or water particles in the air, mixed surface conditions, or fast motion. Moreover, the implementation of an algorithm to define the threshold values has to be performed. This should include the definition of the boundaries for each zone, for instance by the statistical analysis of the groups of points over a huge number of acquisitions. Finally, the development of electronics able to automatically process the measured data and provide an alert signal linked to the state of the surface will be required.

## 5. Conclusions

We proposed an optical method for the detection of dangerous road conditions based on the measurement of the polarization state of the scattered radiation from an asphalt surface by two photodiodes that detected two orthogonal polarized light components. Two main geometries were investigated. The first measured the reflected light, while the second measured the backscattered radiation. In both configurations we also evaluated the need for a third photodiode that measured the total unpolarized scattering. The backscattering geometry was the most reliable, allowing for discrimination among dry, wet, water and ice surfaces, and thus distinguishing the safe condition from the other three different danger levels. In addition, in this case the information from the third photodiode were redundant, allowing a compact sensor geometry.

Thus, we demonstrated the feasibility of a contactless sensor that featured reliability, compactness and was low cost due to the usage of commonly-used commercial components. We stress that the method used here is not dependent on the surface under investigation, and thus it is not limited to the automotive sector. In fact, the reference surface can change from case to case, e.g., a metallic surface (rather than asphalt) in the case of ice detection on wind turbines or airplane wings. Thus, the same approach can potentially be applied to any case where different surface conditions must be identified.
